# Congenital genital abnormalities detected during routine circumcision at a South African institution: a retrospective record review

**DOI:** 10.4314/ahs.v18i2.20

**Published:** 2018-06

**Authors:** Kalli Spencer, Idah Mokhele, Cindy Firnhaber

**Affiliations:** 1 University of the Witwatersrand, Urology; 2 Right to Care; 3 University of the Witwatersrand, Clinical HIV Research unit

**Keywords:** Congenital genital abnormalities, circumcision, South Africa

## Abstract

**Background:**

Due to the reduction in HIV transmission through male medical circumcisions (MMC), numerous clinics throughout South Africa offer a voluntary free service to boys from the age of ten years and above. An examination prior to the procedure may detect congenital abnormalities missed after birth.

**Objectives:**

The aim of this study was to measure the incidence of these abnormalities, determine the demographic and clinical characteristics of this group and determine what referral systems, interventions, and follow-up is available to them.

**Methods:**

The study was a descriptive, observational, retrospective analysis of de-identified medical records at a routine MMC service at a Johannesburg clinic in 2015. The participants were male patients between the ages of 10 – 49.

**Results:**

Out of 1548 participants, 91.0% (n=1409) had a normal genital examination while 3.7% (n=57) had an abnormal examination and 5.1% (n=79) had no examination recorded. Thirty five congenital anomalies were detected and only 2 patients (diagnosed with hypospadias) were seen at the urology out-patient's department.

**Conclusion:**

The incidence of congenital genital abnormalities of males presenting for routine circumcision is low. Despite the low incidence the effect on fertility, sexuality, ability to urinate and on psychological wellbeing is significant. Referral services to the urology department should be restructured to improve all outcomes.

## Background

Sub-Saharan Africa has the highest incidence of HIV in the world.^1^ Due to the reduction in HIV transmission through male medical circumcisions (MMC), numerous clinics throughout the region offer a voluntary free service to boys from the age often years and above. In South Africa, medical male circumcision (MMC) has been identified as a priority tool in the “Strategic Plan for the Scale up of Medical Male Circumcision, 2012–2016” and since 2010, South Africa has been providing MMC as part of a larger HIV prevention strategy.^2^ Since 2011, the US Agency for International Development (USAID) has supported the South African Department of Health (DOH) to roll-out its national strategy of MMC.

The MMC encounter has many benefits for males, who rarely engage in health services.^3^ These male patients are taught about sexual behaviour and the associated risk. There is also an opportunity for health promotion and screening. The physical exam is done by a trained nurse and any abnormalities are confirmed by a doctor. During this examination congenital abnormalities missed after birth may be detected.^4^,^5^ The patient can then be referred to the appropriate specialist.

In many parts of rural Southern Africa babies are delivered by community level midwives who may lack the necessary and specialist expertise to pick up these congenital defects.^6^ Some commonly screened anomalies include: hypospadias, phimosis, undescended testes (cryptorchidism) and epispadias. Besides the health care workers not detecting these anomalies, the patients are also ignorant to the fact they have a problem. Ozoemena and Mbah^6^ found 44% of respondents at a school ranging from age 9–23 were completely unaware of their anomalies. The level of case ignorance was more prevalent in rural as compared to urban schools (84.6% vs 51.4%) despite the higher frequency of cases found in the former. General awareness scores were found to be significantly higher with increasing age and year of study. They found that some of the reasons for late detection of external male genital defects are “ignorance, religious and cultural inhibitions about sexuality, denial, false beliefs and alternative health care-seeking behaviour.” Low level of awareness is probably related to the poor literacy level of the learners. In a Nigerian study of adult men, awareness about undescended testes was very poor, as only two (11.1%) men had self-diagnosed their condition and sought medical attention after 30 years.^7^

In the developed world, Donaruma-Kwoh^8^ at a children's hospital in Houston, Texas found that paediatric chief residents don't examine the genitalia of their male patients; and have difficulty in diagnosing common abnormalities-with only 22% of the residents being able to detect hypospadias correctly. Identification of congenital abnormalities can therefore be taught to any healthcare provider with the appropriate training and skills set. Osifo & Osaigbovo^7^ recommend that health awareness programs should be established as these will aid in early presentation and prevent the development of irreversible complications.

There is significant benefit in detecting these anomalies. Some of them have an effect on urination, sexual function and psychological function. If uncorrected, some of them could manifest as profound social, psychological, medical, and marital complications in adult life.

The aim of this study was therefore to measure the incidence of congenital genital abnormalities in male patients over ten years of age presenting for routine medical circumcision in an urban MMC clinic in Johannesburg, South Africa. The study also aimed to determine the demographic and clinical characteristics of male patients who had documented congenital genital abnormalities identified during routine medical circumcision and determine what referral systems, interventions, and follow-up was for these patients.

## Methods

The study was carried out as a retrospective record review of de-identified, routinely collected medical records at an MMC clinic and Urology out-patient unit at Helen Joseph Hospital. The MMC clinic is a gateway clinic adjacent to the Helen Joseph hospital which is a “super-speciality” regional government facility. Medical records were screened for inclusion: males between the ages 10–49 years of age, patients presenting for routine medical circumcision at the site between January 2015 and December 2015. Patients were excluded if the file was unavailable and/or the patients were from the local prison facility.

Included records were reviewed and clinical and socio-demographic data were captured from patient medical files. Captured data was analysed according to study objectives.

### Study site

The study site was based at the Helen Joseph Hospital, a secondary level government hospital serving the uninsured population in South Africa, located in Johannesburg, South Africa. Patients identified with anomalies requiring specialist consultation during screening at the MMC clinic were referred to the Urology out-patient unit in the Helen Joseph Hospital.

### Sample size

The sample size was set at a census of all patient entries available for the proposed study period at the study site. The study is descriptive in nature and therefore there is no hypothesis to test, and no corresponding calculations of power. At the time of protocol submission approximately 2634 males were circumcised at the study site during the proposed study period, and therefore, we requested that the sample size be set at 2650.

### Study procedures

RTC uses a cloud-based database, RightMaxTm, to captures financial and programmatic data for the MMC programme at all its MMC clinics including the study site. This system is used for reporting and monitoring purposes for the MMC programme.

### Data collection

**Medical records review:** All data was protected by password on secure computers. The MMC database was exported from RightMax into Microsoft Excel and included basic demographic and MMC services related data. The resulting list of study IDs had their other medical records sought from the MMC clinic and Urology Out-patient Department (OPD) unit for review for the study. Files were searched for in the file records archive and on the hospital computer system.

Study IDs were assigned to each eligible file and reviewed by a trained study team staff member. Patient identifiers were only required to link patients from the MMC clinic to the Helen Joseph Hospital Urology unit (to determine successful referral). Study ID, name, date of birth, and file numbers were captured in the linking file.

The study team reviewed and assessed medical records at the study sites following STROBE guidelines^9^. Study IDs were assigned to each individual whose records were included in the study. Medical record data was entered into the CRF by the study team. Study data was collected and managed using the REDCap (Research Electronic Data Capture) electronic data capture tools hosted at University of Witwatersrand.^10^

### Measurement and analysis

#### Descriptive aim

Descriptive statistics were used to summarise patient demographic and clinical characteristics.The following data fields were collected from the participant medical records: demographics; MMC service provision information; clinical characteristics (including relevant history and genital examination findings -phimosis, paraphimosis, epispadias, hypospadias, irretractable foreskin, unilateral/bilateral palpable testicles, other); and referral information.

Continuous variables were expressed as the mean (standard deviation) and categorical data were expressed as frequencies and proportions.

All statistical analyses for the study were performed using the statistical package Stata version 14 (StataCorp, 2015).

### Ethical considerations

Ethics approval was obtained by Witwatersrand University Research ethics Committee (REC). M160651

## Results

**Figure 1:**
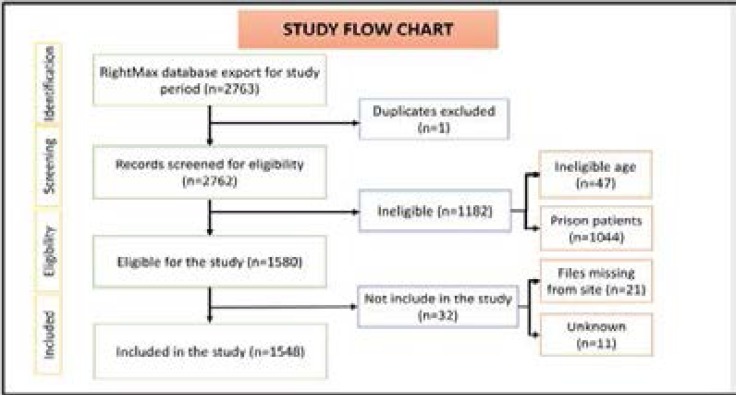
Inclusion and exclusion criteria.

A total of 2762 files were screened. 1548 patients were included in the study and 1214 were excluded based on the following criteria: Age (<10 or >49), prison patients, duplicated files, missing files, unknown (files taken home or not recorded). (Figure 1)

The demographics of the study participants are listed in Table 1.

**Table 1:** Socio-demographic characteristics of study participants (n=1548)

Factor	Category	n (%)
**Age in years**	Median (IQR)	20 (14–28)
	10–17	680 (43.9)
	18–25	380 (24.6)
	26–33	295 (19.1)
	34–41	129 (8.3)
	42–49	63 (4.1)
	Missing	1 (0.1)
**Residence**	City of Johannesburg	1446 (94.7)
	Other	61 (3.9)
	Missing	21 (1.4)
**MMC consent**	Yes	1548 (100)
**MMC Performed**	Yes	1547 (100)
	No (Duplicate file)*	1 (0.1)

* Duplicate file

Out of 1548 participants, 91.0% (n=1409) had a normal genital examination while 3.7% (n=57) had an abnormal examination and 5.1% (n=79) had no examination recorded.

Abnormal examination findings not considered a congenital anomaly are noted in Table 2.

**Table 2:** Pathological genital findings (excluded from study criteria)

Genital condition (n=22)	Number (%)
Adhesions	6 (0.3)
Discharge	3 (0.1)
Ulcers	5 (0.2)
Warts	3 (0.1)
Insufficient skin	1 (0.04)
Skin Tags	1 (0.04)
Unknown	3 (0.1)

**Figure 2:**
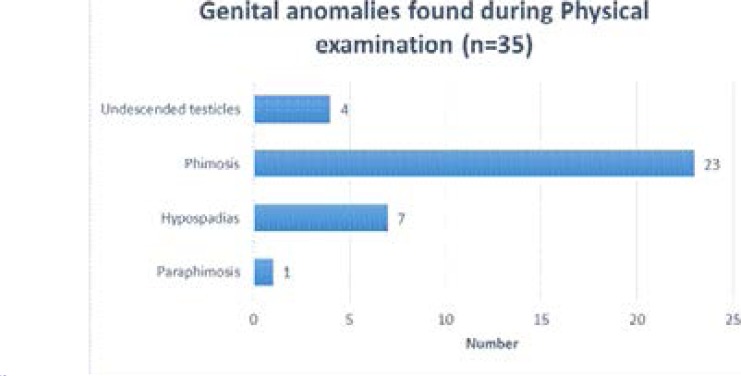
Congenital anomalies Recorded

Thirty five congenital anomalies were regarded as illustrated in Figure 2.

Only 2 out of 35 (5.7%) of the patients who were noted to have congenital anomalies in their files and the referral book were seen at the urology out-patient clinic. Both patients were diagnosed with hypospadias and had surgery arranged.

## Discussion

In this study 7/1548 (0.01%) participants had hypospadias on examination. In a Nigerian study of 6226 subjects ranging from 9 to 23 years, 416 (6.7%) had evidence of defects in their external genitalia. Hypospadias was divided into position of the external urethral meatus: glandular (0.24%), penile (0.10%), peno-scrotal (0.02%).^7^ Turk et al from Turkey^4^ did a pre – circumcision examination in 1,695 patients aged between 6 and 17 years and found hypospadias in 19% of patients. Wan and Wang^11^ examined 2241 male children in 8 Chinese kindergartens and observed a hypospadias incidence of 0.2%. In boys presenting for elective circumcision (age 2 days to 11 years) in Pakistan, 29/318 (9%) had external genital anomalies. Out of 318 patients, 2 had hypospadias.^4^ In a Brazilian study of participants over 40 years of age presenting for routine prostate cancer, there was a 0.6% prevalence of hypospadias.^12^

We found a 0.02% incidence of phimosis in this study. Romero et al found a 0.5% incidence of phimosis.^12^ Wan and Wang noted that 55.5% of children aged 3 to 4 years and 44.1% aged 5 to 6 years were found to have persistent phimosis.^11^ Yesildag in a paediatric out-patient surgery department observed that physiologic phimosis resolves and the foreskin becomes retractable by the age of 3 in almost 90% of boys^5^. Shankar and Rickwood found the incidence of pathological phimosis in boys was 0.4 cases/1000 boys per year, or 0.6% of boys affected by their 15^th^ birthday.^13^

We found 4/1548 (0.003%) had a unilateral undescended testicle. Turk et al, noted an incidence of 13.8%, Wan and Wang a 0.4% incidence and Ozoemena & Mbah a 4.3% incidence.^4^,^11^,^6^ At the age of 1 year, undescended testicles (UDT) in term and / or birth weight >2.5 kg infants was seen in 1.0–1.5%, at 6 years in 0.0–2.6%, at 11 years in 0.0–6.6% and at 15 years in 1.6–2.2% of boys. The actual frequency of acquired UDT essentially remains unclear because of the shortage of studies performed at an older age, and of studies reporting on previous testicular position.^14^

None of our patients presented with the rare epispadias anomaly. Cervellione et al found a 1:117,000 incidence of epispadias while Ozoemena & Mbah noted a 0.02% incidence.^15^,^11^

There are numerous benefits to treating these conditions.^11^ Mureau et al found that males who were dissatisfied with their penile appearance are more at risk for psychosocial problems.^16^ Dorsal chordee (curvature) on erection is an integral part of the exstrophy/epispadias complex. The degree is variable but in most it is a significant handicap to sexual intercourse. Epispadias and hypospadias can both result in slow ejaculation, erectile deformity and social ostracism.^17^ Clinicians treating children with genital anomalies must be aware of the psychiatric risks and vulnerabilities. Screening all children with genital anomalies for psychosexual problems at various developmental stages would be beneficial.

Very few of our participants visited the urology service despite being referred. This may be due to poor communication; reduced awareness of patient or health care worker; inconvenience; anxiety or fear; or economic reasons. A well-structured referral system will make the process more efficient. A recommendation could be based on that by Straus et al who developed a digital online eReferral system which is adaptable, easy to use and assured that few patients were lost to follow up.^18^ Education and awareness of staff members and patients is essential. Buy in from nursing and administrative staff and relevant stakeholders would be crucial to its success.

Dedicated health care workers, also known as patient navigators, could also be introduced to facilitate the process. Their function is to “take individual patients through the continuum of health care as it pertains to their disease, ensuring that any and all barriers to that care are resolved.” In this way patients are “linked” to relevant services and “retained” in the system.^19^

## Limitations and strengths

This study detected a lower incidence of congenital genital anomalies compared to other studies. This could in part be due not only to the retrospective nature of the study but also limited documentation. The recording of genital findings was often done by nurses who may not have the specialized experience or knowledge to detect congenital genital anomalies.

In addition our study was done in a “primary care setting” and the other studies discussed were conducted at surgical and specialist departments possibly leading to a referral bias. This is also a single centre in an urban area of South Africa and therefore may not be fully representative of rural areas or other countries. Another limitation was the clinic and hospital filing systems at times made record retrieval difficult.

The study, however, is unique as it is the first of its kind in South Africa and provides important epidemiological information regarding congenital anomalies. There is also a follow up arm to test system efficiency and propose solutions to improve health out comes. It also highlights the important opportunity these consultations provide with young men who might not have otherwise presented to a health care facility.

## Conclusion

The incidence of congenital anomalies noted in pre- and pubescent boys presenting for routine voluntary medical circumcision is low. An appropriate referral system linked with the circumcision sites nearest urology department will aid with appropriate specialist management of these conditions.
